# Lipids and All-Cause Mortality among Older Adults: A 12-Year Follow-Up Study

**DOI:** 10.1100/2012/930139

**Published:** 2012-05-01

**Authors:** Marcos Aparecido Sarria Cabrera, Selma Maffei de Andrade, Renata Maciulis Dip

**Affiliations:** ^1^Discipline of Geriatrics, Postgraduate Program in Public Health, State University of Londrina (UEL), Robert Koch Avenue, 60 CEP, 86038440 Londrina, PR, Brazil; ^2^Department of Public Health, Postgraduate Program in Public Health, State University of Londrina (UEL), Robert Koch Avenue, 60 CEP, 86038440 Londrina, PR, Brazil

## Abstract

This is a 12-year follow-up cohort study with 800 people (60–85 years old). The association between lipid disorders and mortality was analysed by Cox proportional hazard adjusted model. All-cause mortality was considered the dependent variable, and lipid disorders as independent variables: total cholesterol (TC) >200 and <170 mg/dl, HDL-c <35 and 40, LDL-c >100 and 130, and triglycerides (TG) >50. An initial analysis of all subjects was performed and a second was carried out after having excluded individuals with a body mass index (BMI) <20 kg/m^2^ or mortality in ≤2 years. The mortality showed a positive association with low TC and a negative association with high TC and high LDL-c. After the exclusion of underweight and premature mortality, there was a positive association only with TC <170 mg/dl (HR = 1.36, CI95%: 1.02–1.82). The data did not show a higher risk with high levels of TC, LDL-c, and TG. However, they showed higher mortality among older adults with low TC.

## 1. Introduction

Dyslipidemias, particularly hypercholesterolemia, hypertriglyceridemia, and low high density lipoprotein cholesterol (HDL-c), are known as important risk factors for cardiovascular morbidity and mortality among the population in general [1] and have recently been associated with some chronic degenerative diseases such as dementias [2].

 However, the role that lipid disorders play as mortality risk factors in older adult populations is not very clear [3], even though they present the highest morbidity and mortality rates.

There is some evidence that the relation between total cholesterol (TC) and all-cause mortality varies according to age. Kronmal et al. suggested that although there is a positive relation (high TC and high mortality) at age 40, the direction of the risk becomes negative by age 80 (high TC and low mortality) [4]. This inversion of high TC risk in very old people has been confirmed by other authors [5].

Low TC has been identified as a risk factor for older adult mortality but is commonly attributed to the fact that low TC levels indicate malnutrition, chronic infections, and subclinical or hidden diseases [6, 7].

The objective of this study was to analyze the risk of death from lipid disorders in older people up to 85 years of age over a 12-year follow-up period.

## 2. Materials and Methods

### 2.1. Participants and Setting

Observational prospective cohort study with 800 individuals between 60 and 85 years old who were treated at two geriatric clinics in the city of Londrina, PR, Brazil. Baseline: June/1997 to July/1998; end point: march 2010. Those with diagnosed active neoplastic disease (except skin and prostate neoplasm) or difficulty standing for the anthropometric measurements were excluded beforehand.

### 2.2. Measurements at the Baseline Examination

The variables gender, age, and smoking were evaluated, as well as the presence of diabetes, hypertension, and previous cardiovascular disease (heart failure, coronary diseases, arrhythmias, pacemaker, myocardial infarct, and stroke). Height and weight were measured and body mass index was calculated (BMI).

Serum lipoprotein levels (total cholesterol, HDL-c and triglycerides) were measured using standard enzymatic techniques. Based on this information, the following variables were calculated: non-HDL-c cholesterol (total cholesterol—HDL-c) and low-density lipoprotein cholesterol (LDL-c) [8]. Regarding analysis of LDL-c, 14 elderly people with triglycerides >400 mg/dL were excluded.

Lipid alterations were classified and analyzed according to the following values [1]:

- total Cholesterol (TC) ≥200 mg/dL,
- total cholesterol (TC) <170 mg/dL,
- high-density lipoprotein cholesterol (HDL-c) <40 mg/dL,
- high-density lipoprotein cholesterol (HDL-c) <35 mg/dL,
- low-density lipoprotein cholesterol (LDL-c) >130 mg/dL,
- low-density lipoprotein cholesterol (LDL-c) >100 mg/dL,
- cholesterol non-HDL-c (non-HDL-c) >170 mg/dL,
- triglycerides (TG) >150 mg/dL,
- triglycerides (TG) >200 mg/dL.

### 2.3. Followup

During the entire the followup period, the older adults were monitored every six months by means of either phone calls or medical appointment.

 Individuals who were not found after an active search using addresses, health insurance, retirement, and mortality data were considered as losses and censured in the analysis model subsequent to the last followup contact date.

### 2.4. Statistical Analysis

Data were stored and analyzed using the program EPINFO—version 3.5. The significance level was set at 5%. The chisquare test was used to compare the sample characteristics according to gender for categorical variables. Analysis of variance (ANOVA) was used to compare numerical variables with homogenous variances; otherwise the Kruskal-Wallis test was used.

 Cumulative survival probability was computed using Cox proportional hazards modeling; the outcome analyzed was all-cause mortality and the independent variables were dyslipidemias, which were presented as categorical variables. The regression model was adjusted for gender, age range >75 years old, hypertension, and diabetes.

A second analysis was carried out with a model adjusted for gender, age range, hypertension and diabetes, but excluding the older people who died in the first two years or whose BMI dropped to <20 kg/m^2^.

 The purpose for excluding patients who died prematurely or became underweight was to reduce the impact of preexisting diseases as the cause of death and to minimize the interference of low weight on cholesterol levels, malnutrition and mortality.

### 2.5. Ethical Aspects

This study was approved by the Ethics Committee of the Universidade Estadual de Londrina, and all patients provided written informed consent at the beginning of followup.

## 3. Results

The sample consisted of 800 individuals from 60 to 85 years old (mean age = 71.2; median = 70.5) and was predominantly female (66.7%). Mean followup time for those whose outcome was not presented was 146.6 months. Followup with 38 people was lost (4.8%).

 Three-hundred thirty-nine deaths were registered (42.9%), being more frequent among men (48.9%). Mean TC, non-HDL-c, HDL-c, and LDL-c were higher among women. High blood pressure was more frequent among women and smoking was more frequent among men (Table 1).

In the Cox proportional hazard analysis adjusted for gender, age range >75 years, hypertension, and diabetes, there was a positive association between mortality and the total cholesterol <170 mg/dL variable. The variables total cholesterol >200 mg/dL, cholesterol non-HDL >170 mg/dL, and LDL-c >130 and >100 mg/dL were negatively associated with mortality (Table 2).

After excluding 116 individuals who died prematurely (<2 years) or were underweight (BMI <20 kg/m^2^), the only variable presenting positive association with mortality was total cholesterol <170mg/dL (*P* = 0.04). In this stratified analysis, no variable presented a statistically significant negative association with mortality (Table 3).

## 4. Discussion

The results indicate higher mortality among older people with lower levels of total cholesterol. Furthermore, they show no association between all-cause mortality and hypercholesterolemia, high LDL-c, low HDL-c, hypertriglyceridemia, and high non-HDL-c in this group of older adults.

 High levels of lipoproteins such as TC, LDL-c, and TG are widely known as risk factors for total and cardiovascular mortality in the general population [1]. In geriatric populations, however, this association is being studied, and conflicting results have been presented. These results depend on methodological characteristics such as age, follow-up time, and the covariables analyzed [7–11].

 Our results did not show a positive association between hyperlipidemias and all-cause mortality. In the initial analysis, even before excluding premature mortality and underweight individuals, this association was negative.

 Questions persist about the real role of hypercholesterolemia as a general mortality risk among older people; the association may be positive, negative, or null. The fact that other authors have not observed a positive association corroborates our results [8, 9, 11].

Concerning the risk of developing cardiovascular disease, there is more evidence of the role of TC increase in the geriatric population [12, 13]. It is possible that among elderly populations, high TC levels are associated with better global health conditions. In a study carried out in Honolulu, researchers observed that high TC levels were associated with higher body mass index levels, high HDL-c, better hemoglobin levels, and greater muscular strength [7].

Another factor that could influence analysis of lipid risk factors in older people is cardiovascular mortality before age 60 among individuals with high cholesterol.

In addition to not showing a mortality increase with high liproprotein levels, data from the present study indicate that there is a higher risk in low lipoprotein levels such as TC and HDL-c.

 Low HDL-c levels have already been established as determinants of cardiovascular morbimortality among geriatric populations [14, 15]. In this study, we have observed that even using two different HDL-c cut-offs (<35 and 40 mg/mL) [1], there was positive association with all-cause mortality, although not statistically significant. This corroborates the results of other authors who have observed a similar association in samples of people aged 65 or older [16], 70 or older [17], and in the frail elderly [18].

 Low TC levels represented an important mortality predictor in our analysis of 800 older people (HR = 1.60; CI95% 1.26–2.04). Low TC is related to nutritional deficiencies and poor general health, which could justify this poor prognostic.

Nevertheless, the association remained positive in a second analysis after exclusion of individuals with BMI <20.0 kg/m^2^or of cases that died in the first two years. Persistence of risk even after these exclusions could suggest that low-serum TC represents a poor prognostic indicator independent of association with subclinical or hidden diseases and even nutritional deficiency, which by itself would represent an important risk factor in this age range.

The role of low cholesterol as a risk marker of mortality among older people has also been analyzed by other authors who used different methodologies. Tuikkala et al. analyzed and followed up a group of home-dwelling elderly persons for six years and found a positive association between low TC and all-cause mortality, independently of comorbidities and general health condition [19]. Like our results, this data reinforces the role of low cholesterol as an indicator of higher mortality risk without necessarily being associated with previous clinical situations.

This possibility has been reinforced by another study that suggested low TC as an independent mortality predictor in the elderly. Even though their sample was small, the authors demonstrated that after the exclusion of patients with acute diseases, dementia, infection, and malnutrition, low TC was associated with higher mortality within a 2-year period [20].

Positive association between low TC and all-cause mortality was also identified among elderly Italians of an age range similar to that of the participants in the present study (mean of 73 years, excluding those 85 or older), but with shorter follow-up time (3 years) [21].

Also, within a short three-year follow-up period, nondemented older people with lower TC, non-HDL-c and LDL-c levels had a higher mortality risk, even after adjusting for heart diseases, smoking, and diabetes. Levels of association with mortality were no different among those older than 75, and this association was mitigated by the exclusion of mortality in the first year [9].

Volpato et al. showed higher mortality risk with low TC and suggested the inclusion of serum albumin and HDL-c values in the clinical evaluation to enhance the risk represented by low TC [22].

 Akerblom et al. analyzed a group of older people for a 12-year follow-up period and identified that low TC, non-HDL-c, and LDL-c levels were associated with higher mortality among white and black subjects but not among Hispanics [11]. The lipid cutoff levels in their study were similar to those used in the present analysis.

In general, the values used to describe low TC levels were similar in all the studies presented (160 to 180 mg/dL); in the present study, we used the cut-off <170 mg/dL, which corresponded to that used by other authors.

Some methodological characteristics of this study deserve attention for appropriate extrapolation of the data obtained in this analysis. Due to the long follow-up period, it was possible to observe outcomes after many years, reducing the possibilities of events having happened due to previous disease situations. The exclusion of subjects older than 85 in a follow-up study is necessary to minimize the effects of expected high mortality of very old people during the time of the study.

It is important to highlight some limitations that could have influenced the results of this analysis such as non-acknowledgment of the use of lipid-lowering drugs, other comorbidities, and smoking.

In spite of the fact that the sample was composed by outpatients, some demographic, lifestyle, and comorbidities characteristics are similar to those of the general elderly population. Therefore, it seems reasonable to extrapolate these results to larger older populations.

Based on what we observed, we conclude that the reduction of TC levels can be interpreted as an indirect risk marker of morbidity and mortality in apparently healthy older individuals.

Therefore, low or declining total cholesterol tests could be used in clinical practice for decision making regarding further nutritional investigation as well as to track hidden diseases such as malignant neoplasms. However, the results of this study suggest that, even without such aggravating factors, older people with low TC represent a more vulnerable group.

Another aspect to be considered in clinical practice is the pertinence of pharmacological measures for reducing TC levels in primary and secondary prevention of cardiovascular diseases among this age group.

It is necessary to carry out further studies that contribute toward understanding the factors related to decreased TC and their impact on health risks in the geriatric population. Similarly, it is also necessary to reevaluate strategies for clinical control of dyslipidemias in older patients.

##  Conflict of Interests

The authors declare any conflicts of interest that may be inherent in their submissions.

## Figures and Tables

**Table 1:** Characterization of older people according to sex.

	Total (*n* = 800)	Men (*n* = 266)	Women (*n* = 534)	*P*
Age (mean)—years	71.2	70.9	71.3	ns
Hypertension (%)	53.9	47.4	57.1	<0.05
Diabetes (%)	14.9	14.2	15.2	ns
Previous cardiovascular diseases (%)	17.1	19.8	15.7	ns
Smoking (%)	7.6	12.8	5.1	<0.001
BMI (kg/m^2^)	25.9	25.0	26.4	<0.001^#^
Total cholesterol (mg/dL)	205.8	194.7	211.2	<0.001
HDL-c (mg/dL)	49.9	44.8	52.4	<0.001
LDL-c (mg/dL)	126.6	121.1	129.3	<0.05
Cholesterol non-HDL-c (mg/dL)	155.7	149.9	158.6	<0.05
Triglycerides (mg/dL)	146.5	144.0	147.8	ns
All-cause mortality (%)	42.4	48.9	39.1	<0.05

BMI: body mass index; HDL-c: high density lipoprotein cholesterol; LDL-c: low density lipoprotein cholesterol; ns: nonsignificant; ^#^Kruskal-Wallis test.

**Table 2:** All-cause mortality risk and lipid levels in adjusted by sex, age >75, hypertension, and diabetes.

Variable	Hazard ratio	Confidence interval 95%	Value of *P*
Total cholesterol >200 mg/dL	0.76	0.61–0.95	<0.05
Total cholesterol <170 mg/dL	1.60	1.26–2.04	<0.001
HDL-c <40 mg/dL	1.20	0.93–1.54	ns
HDL-c <35 mg/dL	1.21	0.90–1.63	ns
Cholesterol non-HDL >170 mg/dL	0.78	0.61–0.99	<0.05
LDL-c >100 mg/dL	0.69	0.55–0.87	<0.05
LDL-c >130 mg/dL	0.73	0.58–0.92	<0.05
Triglycerides >150 mg/dL	0.82	0.65–1.03	ns
Triglycerides >200 mg/dL	0.81	0.59–1.11	ns

HDL-c: high density lipoprotein cholesterol; LDL-c: low density lipoprotein cholesterol.

ns: nonsignificant.

**Table 3:** All-cause mortality risk and lipid levels in adjusted by sex, age > 75, hypertension, and diabetes (excluded body mass index <20 kg/m^2^ and early mortality <2 years).

Variable	Hazard ratio	Confidence interval 95%	Value of *P *
Total cholesterol >200 mg/dL	0.84	0.65–1.09	ns
Total cholesterol <170 mg/dL	1.36	1.02–1.82	<0.05
HDL-c <40 mg/dL	1.28	0.97–1.70	ns
HDL-c <35 mg/dL	1.34	0.96–1.85	ns
Cholesterol Non-HDL >170 mg/dL	0.88	0.67–1.16	ns
LDL-c >100 mg/dL	0.83	0.63–1.10	ns
LDL-c >130 mg/dL	0.81	0.62–1.05	ns
Triglycerides >150 mg/dL	0.89	0.68–1.16	ns
Triglycerides >200 mg/dL	0.86	0.60–1.23	ns

HDL-c: high density lipoprotein cholesterol; LDL-c: low density lipoprotein cholesterol; ns: nonsignificant.
